# Physiological and Morphological Responses of *Ischaemum rugosum* Salisb. (Wrinkled Grass) to Different Nitrogen Rates and Rice Seeding Rates

**DOI:** 10.1371/journal.pone.0098255

**Published:** 2014-06-09

**Authors:** Tahir Hussain Awan, Bhagirath Singh Chauhan, Pompe C. Sta. Cruz

**Affiliations:** 1 Weed Science, Crop and Environmental Sciences Division, International Rice Research Institute (IRRI), Los Baños, Philippines; 2 Crop Science Cluster, Department of Agronomy, College of Agriculture, University of Philippines, Los Baños, Philippines; University of Vigo, Spain

## Abstract

*Ischaemum rugosum* is a competitive weed in direct-seeded rice systems. Developing integrated weed management strategies that promote the suppression of weeds by crop density, cultivar selection, and nutrition requires better understanding of the extent to which rice interferes with the growth of this weed and how it responds to resource limitation due to rice interference. The growth of *I. rugosum* was studied when grown with four rice seeding rates (0, 25, 50, and 100 kg ha^−1^) and four nitrogen (N) rates (0, 50, 100, and 150 kg ha^−1^). Compared to the weed plants grown alone, weed tiller number was reduced by 63–80%, leaf number by 68–77%, leaf area by 69–77%, leaf biomass by 72–84%, and inflorescence biomass by 81–93% at the rice seeding rates of 25–100 kg ha^−1^. All these parameters increased with increasing rates of N from 0 to 150 kg ha^−1^. At weed maturity, *I. rugosum* plants were 100% taller than rice at 0 kg N ha^−1^, whereas, with added N, the weeds were only 50% taller than rice. Weed biomass increased by 82–160%, whereas rice biomass increased by 92–229%, with the application of 50–150 kg N ha^−1^. Added N favored rice biomass production more than it did the weed. Rice interference reduced the height and biomass of *I. rugosum*, but did not suppress its growth completely. *I. rugosum* showed the ability to reduce the effects of rice interference by increasing leaf area, leaf weight ratio, and specific leaf area, and by decreasing the root-shoot weight ratio in comparison to the weed plants grown alone. The results suggest that rice crop interference alone may reduce *I. rugosum* growth but may not provide complete control of this weed. The need for integrated weed management practices to effectively control this weed species is highlighted.

## Introduction

Majority of the people living in Asia consume rice as their staple food and 90% of the world's rice is produced and consumed in Asia. Here, the major rice establishment method is manual transplanting of seedlings. However, this method is more laborious and requires more water [1]. Shortage of labour and water compels researchers and growers to develop alternative methods of crop establishment, such as dry-seeded rice (DSR). Acceptance of DSR is increasing among farmers in South and Southeast Asian countries. DSR is a resource-conserving technology compared with puddled transplanted rice, except that it is prone to heavy weed infestation [1]. Weeds are principal biotic constraints to rice production in DSR [2] With DSR, rice and weeds emerge approximately at the same time and, therefore, greater effort is needed to control weeds. Manual hand weeding is very expensive, time-consuming, and, sometimes, it is not feasible. Therefore, farmers have to rely mainly on herbicides. Non-judicious use of herbicides, however, is linked with the evolution of herbicide resistance in weeds and concerns over soil and environmental pollution [3], [4].

Efficient weed control in DSR is still a main concern, and strategies are needed to reduce the weed problem. If there is an interest in reducing reliance on herbicides, additional weed management tools (i.e., use of competitive cultivars, high seeding rates, and narrow row spacing) are needed to achieve sustainable weed control [2], [5]. Comprehension of weed biology and ecology is important to develop cultural weed management strategies.

*Ischaemum rugosum* Salisb. is a noxious weed in at least 26 countries of the world [6] and to many crops, including rice[7]. This weed is adapted to a wide variety of habitats. However, there is a scarcity of information in literature on the biology and ecology of this weed. It can emerge even when buried at a 10-cm depth [8] because of its long coleoptile length. At harvest time, it contaminates rice seeds because it has a similar size and shape [9]. *I. rugosum* has a high level of seed dormancy because of the presence of glumes, which delay seed germination after shedding. Therefore, its control by herbicides is difficult in rice. *I. rugosum* causes considerable yield losses in rice—by 50% [7] to 60% [9]. Because of the continuous use of herbicides, this weed has developed multiple resistance to herbicides belonging to different modes of action, that is, ACCase inhibitors, ALS inhibitors, urease, and amides [10]. Thus, nowadays *I.rugosum* has become a serious weed of rice.

Several researchers have projected the use of high rice seeding rates in DSR to suppress weeds and to achieve high rice yield [4], [11]. In an earlier study, weeds severely reduced yield (71%) at a low seeding rate (40 kg ha^−1^), whereas high seeding rates of 80–160 kg ha^−1^ produced high rice yield and minimized losses caused by the weeds [12]. At high seeding rates, the crop may need more nutrients to produce high yield. The effect of N fertilizer on the *I. rugosum-*rice competitive interaction has not yet been studied in Asia. Crop-weed interference can be affected by fertilizer management and seeding rates, and N is one of the crucial components for crop-weed competitive interactions [13]. Some weeds consume high quantities of N, reduce crop N uptake, and suppress growth, biomass, and yield of rice [14]. Other researchers claimed that high doses of N fertilizer enhanced crop growth and yield compared with weeds, and that weed response to added N decreased when they are under shaded conditions [15], [16]. Some scientists found that increasing N rates had little effect on crop-weed competition [17]. Therefore, the effect of N may be species-specific [18], [19]. The effect of crop seeding rate on weed suppression could be affected by N rates. Effects of high crop seeding rate could be more prominent at low N levels because weeds grow slowly in that condition [19].

Weed control practices that enhance the competitive ability of crops over weeds should be a fundamental part of an integrated weed management (IWM) strategy. Before IWM strategies that rely on crop competitiveness can be improved, there is a need to better understand how rice interferes with weed growth, and how weeds compete with rice for resource use. Therefore, a study was conducted to evaluate the physiological and morphological responses of *I. rugosum* to different N and rice seeding rates.

## Materials and Methods

Seeds of *I. rugusom* were collected in 2011 from upland rice fields around Los Baños, Philippines. The first two authors work at this nistitue, and thus no permission was needed to collect the weed seeds. They also confirmed that the study did not involve endangered or protectd species.

Test rice variety is NSICRc222 (IR154). Experiments were conducted in a screenhouse at the International Rice Research Institute (IRRI), Los Baños, Philippines. The screenhouse is made up of a large iron steel frame covered with a 2-mm steel mesh from all sides to maintain environmental conditions similar to field conditions.

The experiments were conducted by growing the weed and rice plants in plastic pots [20], [21] with holes at the bottom filled with sieved soil (8.3 kg pot^−1^). The pots were 25 cm in diameter and 30 cm in height. The soil was sieved through a 3-mm aperture and it was analyzed before use in the experiments. The soil had 22% sand, 38% silt, 40% clay, a pH of 6.0, 0.99% organic carbon, 0.12% Kjeldahl N, 43 mg kg^−1^ soil of available P_2_O_5_, and 1.26 meq 100^−1^ g soil of available K.

In this study, there were 16 treatment combinations of two factors: four N rates—0, 50, 100, and 150 kg ha^−1^—and four seeding rates—0 (0 plants pot^−1^), 25 (5 plants pot^−1^), 50 (10 plants pot^−1^), and 100 kg ha^−1^ (20 plants pot^−1^). Phosphorus and potash fertilizers were applied at sowing at 40 kg P_2_O_5_ ha^−1^ and 40 kg K_2_O ha^−1^, respectively. N in the form of urea was applied in two equal splits at 20 and 40 days after sowing (DAS). Two to three weed seeds were planted at the centre of each pot and covered with a thin layer (2 mm) of soil. In the case of five rice plants per pot (i.e., 25 kg seed ha^−1^), rice seeds were planted in a single circle around the weed plant at a distance of 5 cm. In the case of 10 and 20 rice plants per pot, the plants were planted in two circles. The placement of the first circle was the same as described above and the distance of the second circle was 2.5 cm away from the first circle. In each circle, rice seeds were planted at an equal distance from each other. At 7 DAS, thinning was carried out to maintain the required density of rice and weed plants per pot. Only one plant of *I. rugosum* was maintained at the centre of each pot. A randomized complete block design with three replications was used to arrange the pots. Weeds other than *I. rugosum* were removed manually, as and when required. The pots were placed at a distance of 30 cm from each other to avoid the effect of shading and, fortnightly, pots were rotated to new positions to reduce experimental errors. Pots were irrigated 2–3 times a day with a sprinkler system.

The study was conducted two times with a gap of two and a half months in-between. The first and second experiments began on 4 May and 13 July 2012, respectively. These experiments were harvested on 30 July and 5 October, respectively. Plant height, number of leaves per plant, number of tillers per plant, and SPAD values were measured at 14, 28, 42, 56, 70, and 84 DAS. Plant height of the weed and rice plants were measured from the ground to the tip of the longest leaf.

**Figure 1:**
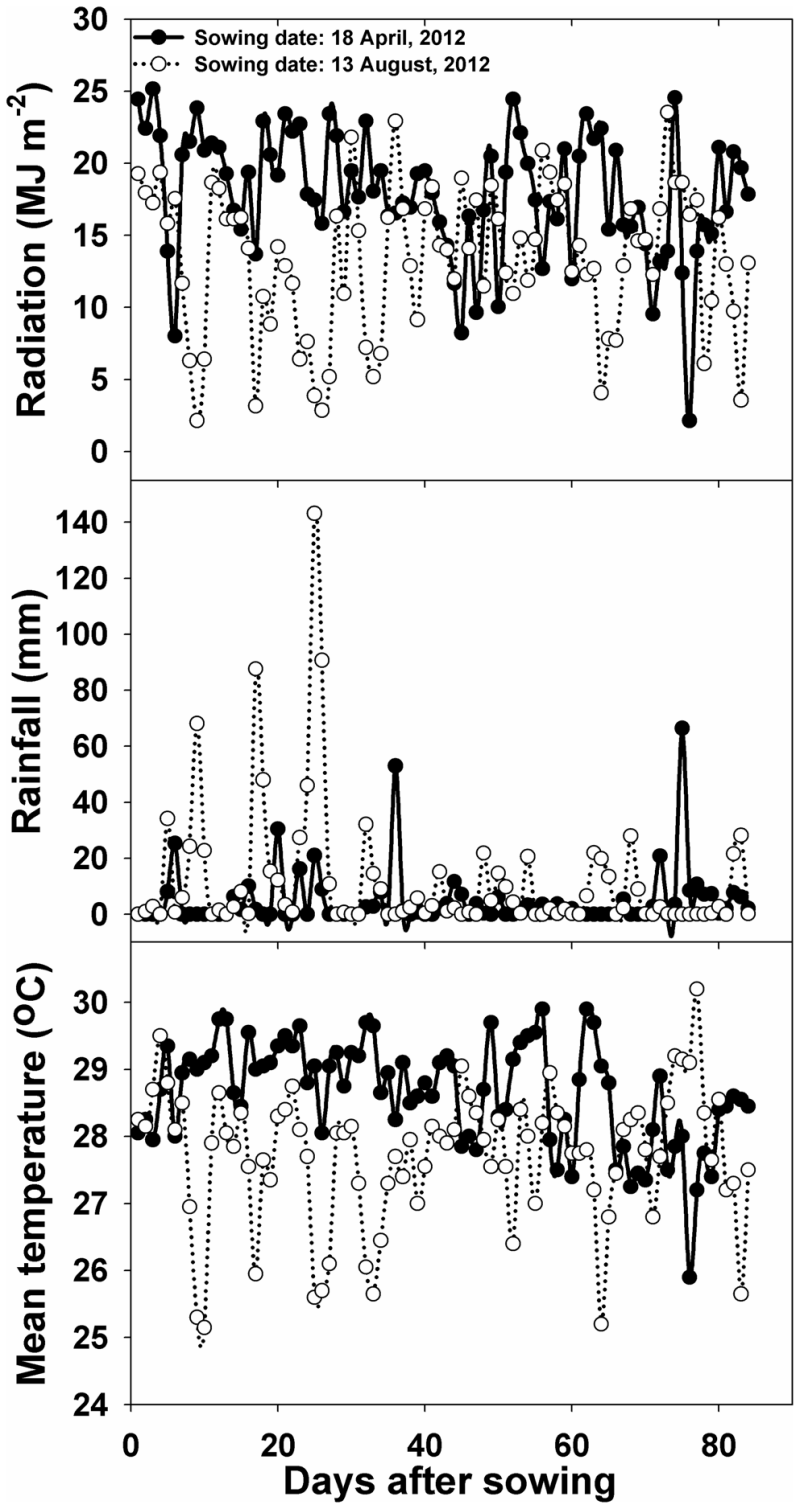
(a) Radiation (MJ m-2), (b) rainfall (mm), and (c) average temperature during the experimental period.

Plants were harvested at weed maturity from the ground level at 84 DAS, with the criterion of weed seed maturity. After detaching leaves for leaf area measurements, shoots were separated into stems and inflorescence. Leaf area was measured using a leaf area meter (LI-COR, model LI-3100, USA). After measuring the leaf area, stem, leaves, and inflorescence were placed separately in paper bags and dried in an oven at 70°C for 72 hours to achieve dry biomass. From each pot, roots were removed with the soil intact, then, were washed through a steel strainer. Weed and rice roots were separated and placed in paper bags for oven-drying and biomass measurements. The following ratios were calculated: root to shoot weight ratio (RSWR), specific leaf area (SLA), leaf weight ratio (LWR), and leaf area ratio (LAR). LAR is the ratio of leaf area to total plant (above- and belowground) biomass and it indicates how photosynthates are being partitioned within the plant parts.
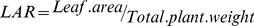
(1)

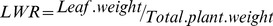
(2)

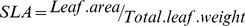
(3)

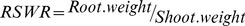
(4)Radiation (MJ m^−2^), rainfall (mm), and mean temperature (°C) during the study period for both experiments are shown in Figure 1.

### Statistical analyses

Data from both experiments were subjected to analysis of variance (ANOVA) (GenStat 8.0, 2005). Data variance was visually inspected by plotting residuals to confirm homogeneity of variance before statistical analysis. The ANOVA results indicated that there were significant interactions between treatments and experimental ‘runs’; therefore, the data were separately analyzed for both experimental runs. Treatment means for SPAD, LAR, LWR, RSWR, and SLA were separated using standard error of difference (SED) at a 5% level of significance. Data were analyzed using regression analysis for plant height, and leaf and tiller numbers per plant of *I. rugosum* and a three-parameter sigmoid model was used:
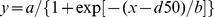
(5)Where: *y* is the estimated plant height, leaf number, or tiller number per plant of *I. rugosum* as a function of rice seeding rate or DAS at time *x*; *a* is the maximum of the parameter; *d50* is the time to reach 50% of the final plant height, leaf number, or tiller number per plant; and *b* is the slope. The data of leaf area and leaf, stem, inflorescence, and total aboveground biomass (g plant^−1^) were fitted to a three-parameter exponential model:
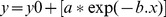
(6)Where: *y* is the leaf area and leaf, stem, inflorescence, and total aboveground biomass (g plant^−1^) at seeding rate *x*; *y0* and *a* are the constants; and *b* is the slope. Root biomass (g plant^−1^) was fitted to an exponential model:
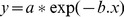
(7)Where: *y* is the estimated root biomass (g plant^−1^) at seeding rate (kg ha^−1^) *x*; *a* is the constant; and *b* is the slope. *R^2^* values were used to determine goodness-of-fit for the selected model. Parameter estimates were compared using their standard error.

## Results

Analysis showed that there were interactions (p<0.05) among experimental run, seed rate, and N rate. Interactions were also significant for experimental run by N rate and experimental run by seed rate for most parameters. Therefore, both experiments were examined separately for N and effects of seed rate.

### Plant height

**Figure 2:**
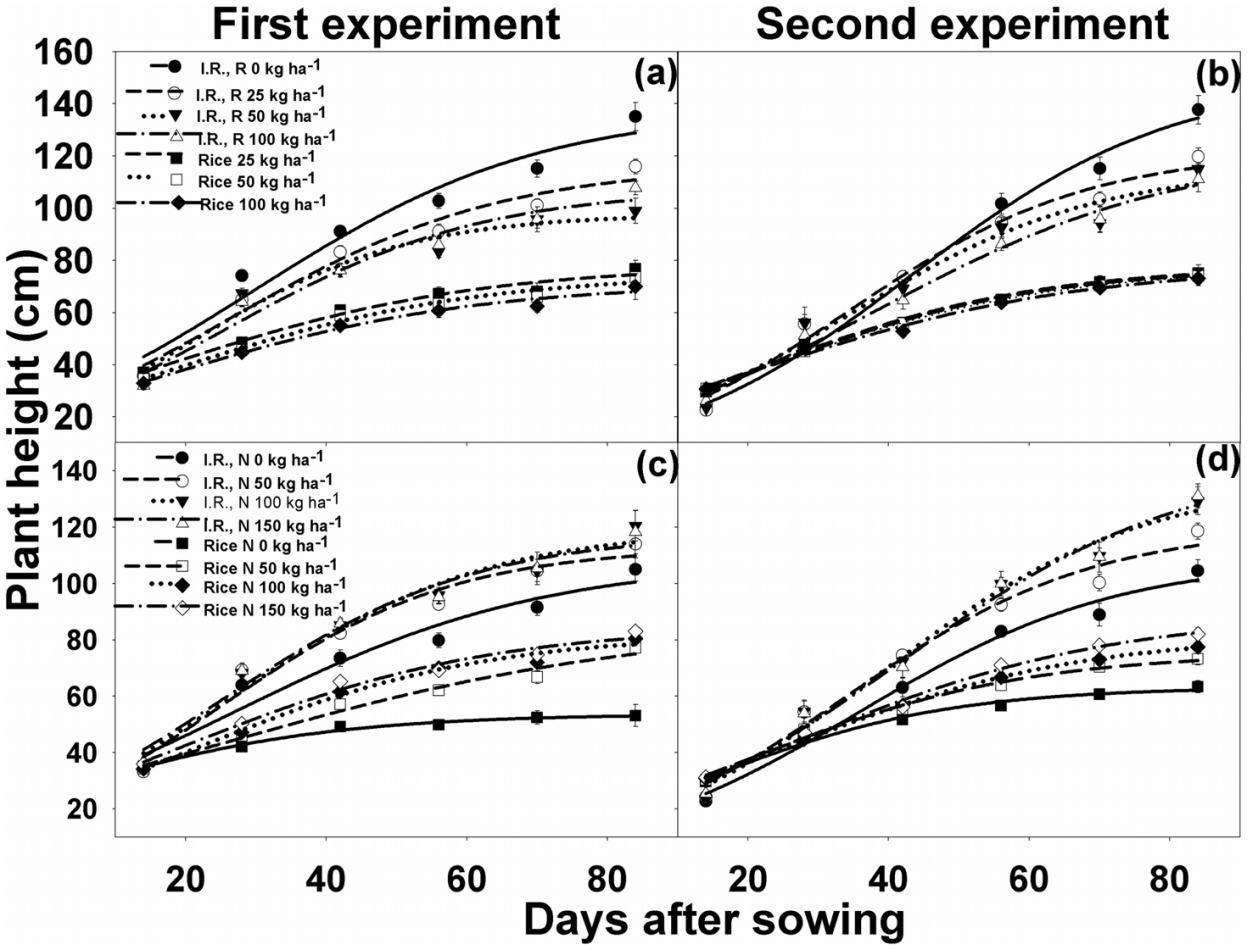
(a, b) Plant height of *Ischaemum rugosum* (I.R.) when grown alone (I.R., R0) or in competition with rice at different seeding rates (R), i.e., 25 (I.R., R25), 50 (I.R., R50), and 100 kg ha^−1^ (I.R., R100); Rice plant height at different seeding rates: 25 kg ha^−1^, 50 kg ha^−1^, and 100 kg ha^−1^; and (c, d) *Ischaemum rugosum* (I.R.) and rice plant height under different nitrogen levels, i.e., 0 (I.R., N0, and rice N0), 50 (I.R., N50, and rice N50), 100 (I.R., N100, and rice N100), and 150 kg ha^−1^ (I.R., N150, and rice N150). The vertical bars represent standard error of means. The lines represent a three-parameter sigmoid model (*y* = *a*/{1+e^[−(*x*−*x*50)/*b*]^}) fitted to the data. Parameter estimates are shown in Table 1.

*Ischaemum rugosum* and rice were almost of the same height at 14 DAS (Figures 2a–d). After that, the height of *I. rugosum* was significantly affected by increasing the seed rates from 25 to 100 kg ha^−1^, and plants grown alone were taller than plants grown with rice (Figures 2a and b). *I. rugosum* was 117–122, 98–116, and 107–122 cm tall when grown with rice at seeding rates of 25, 50, and 100 kg ha^−1^, respectively; whereas, its height was 138–150 cm when grown alone (Table 1).

**Table 1:** Parameter estimates of a three-parameter sigmoid model (*y* = *a*/{1+e^[−(*x*−*x*50)/*b*]^}, where *a* is the maximum height, leaf number, or tiller per plant; *d50* is the time (days after planting) to reach 50% of final height, leaf number, or tiller; and *b* is the slope at time *x*) fitted to the plant height (rice and weed), leaf number, or tiller per plant of *Ischaemum rugosum* when grown alone (I.R., R0) or in competition with a varying density of rice plants (R25, R50, or R100 kg ha^−1^).

	Plant height							
	First experiment				Second experiment			
Treatments	*a*	*b*	*d50*	*R^2^*	*a*	*b*	*d50*	*R^2^*
I.R., R 0 kg ha^−1^	137.83(16.18)	20.26 (6.56)	29.97 (6.32)	0.96	149.51 (9.47)	18.43 (2.29)	43.41 (3.23)	0.99
I.R., R 25 kg ha^−1^	116.46 (9.50)	19.21 (5.01)	26.60 (4.29)	0.97	121.80 (9.21)	16.94 (3.52)	34.44 (3.86)	0.98
I.R., R 50 kg ha^−1^	97.82 (6.12)	15.20 (4.41)	20.81 (3.34)	0.96	115.69 (12.82)	17.82 (5.38)	33.43 (5.76)	0.96
I.R., R 100 kg ha^−1^	107.44 (9.10)	18.25 (5.28)	25.77 (4.42)	0.97	121.70 (10.80)	21.39 (3.91)	38.23 (4.94)	0.99
Rice 25 kg ha^−1^	78.21 (4.61)	22.50 (5.23)	16.19 (3.05)	0.98	77.61(2.95)	19.60 (2.76)	21.74 (1.98)	0.99
Rice 50 kg ha^−1^	74.81 (4.10)	21.75 (4.67)	17.32 (2.84)	0.98	77.29(0.87)	20.59 (0.82)	21.84 (0.59)	0.99
Rice 100 kg ha^−1^	71.19 (3.67)	22.13 (4.50)	16.65 (2.67)	0.98	77.62 (3.90)	22.47 (3.69)	22.08 (2.67)	0.99
I.R., N 0 kg ha^−1^	108.22(15.21)	22.25 (8.90)	26.67 (7.69)	0.95	108.59 (7.62)	18.36 (3.19)	35.78 (3.66)	0.99
I.R., N 50 kg ha^−1^	113.03 (8.04)	16.60 (4.43)	25.05 (3.69)	0.97	120.19 (9.62)	17.52 (3.79)	34.10 (4.13)	0.98
I.R., N 100 kg ha^−1^	119.43 (9.64)	17.99 (4.89)	26.57 (4.21)	0.97	137.05 (11.84)	18.57 (3.58)	39.02 (4.50)	0.99
I.R., N 150 kg ha^−1^	117.81 (8.23)	17.97 (4.41)	25.27 (3.64)	0.97	143.99 (15.02)	20.39 (4.09)	41.61 (5.64)	0.99
Rice N 0 kg ha^−1^	53.69 (1.16)	18.72 (3.26)	2.48 (2.52)	0.99	63.20 (2.05)	16.95 (2.96)	14.85 (1.89)	0.99
Rice N 50 kg ha^−1^	85.74 (10.50)	30.49 (8.79)	24.70 (7.84)	0.98	75.44 (3.30)	19.62 (3.36)	20.14 (2.28)	0.99
Rice N 100 kg ha^−1^	82.23 (3.75)	20.65 (3.42)	21.05 (2.38)	0.99	81.98 (0.62)	21.28 (0.50)	24.54 (0.40)	0.99
Rice N 150 kg ha^−1^	84.84 (4.98)	21.68 (4.59)	19.91 (3.06)	0.98	89.18(4.23)	22.43 (2.95)	27.36 (2.62)	0.99
Number of tillers plant^−1^								
I.R., R 0 kg ha^−1^	20.35 (2.04)	12.19 (3.80)	39.05(4.56)	0.96	20.83(0.34)	8.05 (0.56)	40.73 (0.65)	0.99
I.R., R 25 kg ha^−1^	6.78 (0.93)	16.50 (6.46)	33.74 (6.97)	0.94	7.92 (0.42)	14.45 (2.06)	39.35 (2.52)	0.99
I.R., R 50 kg ha^−1^	8.63 (3.55)	26.67(13.17)	52.26 (25.70)	0.94	6.42 (0.73)	21.39 (3.89)	46.58 (6.22)	0.99
I.R., R 100 kg ha^−1^	8.63 (3.55)	26.67(13.17)	52.26 (25.70)	0.94	6.91 (3.11)	32.07 (10.25)	69.93 (30.18)	0.98
I.R., N 0 kg ha^−1^	5.95 (2.01)	26.81(13.83)	43.59 (21.57)	0.93	6.82 (0.35)	16.17 (2.07)	38.81 (2.53)	0.99
I.R., N 50 kg ha^−1^	10.14 (0.74)	14.77 (2.73)	40.85 (3.45)	0.99	7.79(0.26)	9.74 (1.29)	35.56 (1.50)	0.99
I.R., N 100 kg ha^−1^	11.98 (0.91)	14.32 (2.90)	40.07 (3.59)	0.98	10.80(0.26)	11.23 (0.86)	41.38 (1.05)	0.99
I.R. N 150 kg ha^−1^	11.93 (0.94)	14.25(3.28)	36.97 (3.82)	0.98	13.64 (0.44)	11.13 (1.05)	44.92 (1.34)	0.99
Number of leaves plant^−1^								
I.R., R 0 kg ha^−1^	102.70 (2.18)	10.88 (0.70)	44.08(0.88)	0.99	95.85 (1.75)	9.33 (0.56)	46.39 (0.70)	0.99
I.R., R 25 kg ha^−1^	42.36 (12.25)	25.86 (8.13)	56.17 (17.36)	0.98	48.46 (1.27)	18.82 (0.66)	55.63 (1.26)	0.99
I.R., R 50 kg ha^−1^	39.95 (14.63)	27.02 (9.61)	59.52 (22.37)	0.97	112.80 (53.84)	29.83 (2.90)	112.52 (22.13)	0.99
I.R., R 100 kg ha^−1^					95.61 (247.58)	34.28 (14.9)	124.77 (131.8)	0.98
I.R., R N 0 kg ha^−1^	89.28 (66.67)	39.48 (6.93)	118.27(47.13)	0.99	68.79 (24.22)	29.25 (4.57)	86.43 (19.39)	0.99
I.R., N 50 kg ha^−1^	44.84 (3.15)	14.59 (2.37)	44.44 (3.22)	0.99	46.10 (1.35)	16.23 (0.80)	52.09 (1.32)	0.99
I.R., N 100 kg ha^−1^	61.15 (4.95)	15.41 (2.51)	47.65 (3.69)	0.99	52.45 (2.80)	11.67 (1.62)	47.86 (2.16)	0.99
I.R., N 150 kg ha^−1^	62.62 (2.97)	14.07 (1.57)	45.01(2.13)	0.99	66.46 (2.86)	11.13 (1.21)	50.58 (1.65)	0.99

Nitrogen levels did not affect weed height within 14–28 DAS, although an apparent effect of N was observed within 42 to 54 DAS. Compared to the height at 14 DAS, the rates of increase in height at 84 DAS were 218, 244, 248, and 223% for *I. rugosum* when fertilized with 0, 50, 100, and 150 kg N ha^−1^, respectively. These values for rice were 51, 126, 135, and 130%, respectively. Plant height increased with increasing N rates of up to 100 kg ha^−1^. Beyond this rate, N had no positive effect on the height of *I. rugosum*, whereas it had a positive effect on the height of rice. In the first experiment, the weeds were 49, 31, and 51% taller than rice at seeding rates of 25, 50, and 100 kg ha^−1^, respectively; whereas at 0, 50, 100, and 150 kg N ha^−1^, the weeds were 101, 32, 45, and 39% taller than rice, respectively. A similar trend was observed for the second experiment.

### Tiller density

**Figure 3:**
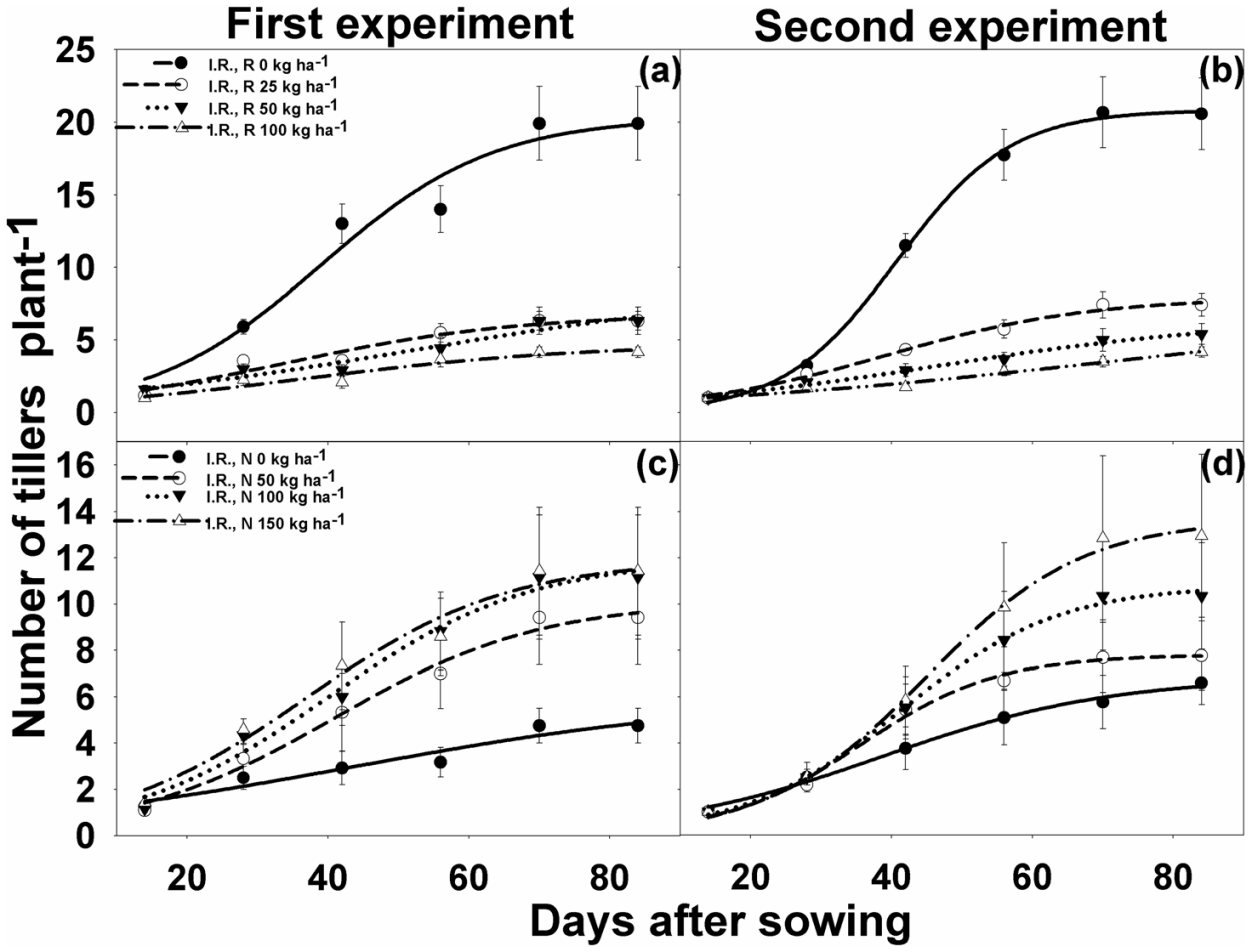
(a, b) Number of tillers per plant of *Ischaemum rugosum* (I.R.), rice seeding rates (R) when grown alone without rice (I.R., R0) or in competition with rice at different seeding rates (R), i.e., 25 (I.R., R25), 50 (I.R., R50), and 100 kg ha^−1^ (I.R., R100); and (c, d) number of tillers per plant under different nitrogen levels, i.e., 0 (I.R., N0), 50 (I.R., N50), 100 (I.R., N100), and 150 kg ha^−1^ (I.R., N150). The vertical bars represent standard error of means. The lines represent a three-parameter sigmoid model (*y* = *a*/{1+e[−(*x*−*x*50)/*b*]}) fitted to the data. Parameter estimates are shown in Table 1.

The number of weed tillers per plant was not affected by varying rice densities until 14 DAS, although, it declined significantly (p<0.001) beyond 14 DAS when grown with rice (Figures 3a and b). At 84 DAS, the maximum number of tillers per plant (parameter *a*) were 20.4 to 20.8, when weed was grown alone. As the rice seeding rates increased from 0 to 100 kg ha^−1^, the number of weed tillers decreased (Table 1). In the first experiment (Figure 3a), rice seeding rates of 25 and 50 kg ha^−1^ had a similar effect and suppressed the tiller number by 67% (6.8 tillers plant^−1^); whereas 150 kg ha^−1^ of seeding rates suppressed tiller density by 79% (4.7 tillers plant^−1^). In the second experiment (Figure 3b), however, all seeding rates suppressed the tiller density significantly beyond 14 DAS. In terms of percent reduction, the tiller number plant^−1^ was reduced by 62%, 74%, and 80% at seeding rates of 25, 50 and 100 kg ha^−1^, respectively (Table 1).

The increased levels of N significantly increased the number of tillers of *I. rugosum* after 14 DAS (Figures 3c and d). In the first experiment, N application at 0 to 100 kg ha^−1^ significantly increased the number of tillers per plant, whereas no significant differences were observed between 100 and 150 kg N ha^−1^. However, in the second experiment, all N levels had a significant effect on tiller density compared with tiller density (6.0 and 6.8 tillers plant^−1^ in the first and second experiments, respectively) without added N. Nitrogen at 50, 100, and 150 kg ha^−1^ increased tiller number by 70% (10.1 tillers plant^−1^), 101% (12 tillers plant^−1^), and 101% (12 tillers plant^−1^), respectively, for the first experiment; and 14% (7.8), 58% (10.8), and 100% (13.6), respectively, for the second experiment (Table 1).

### Leaf number

**Figure 4:**
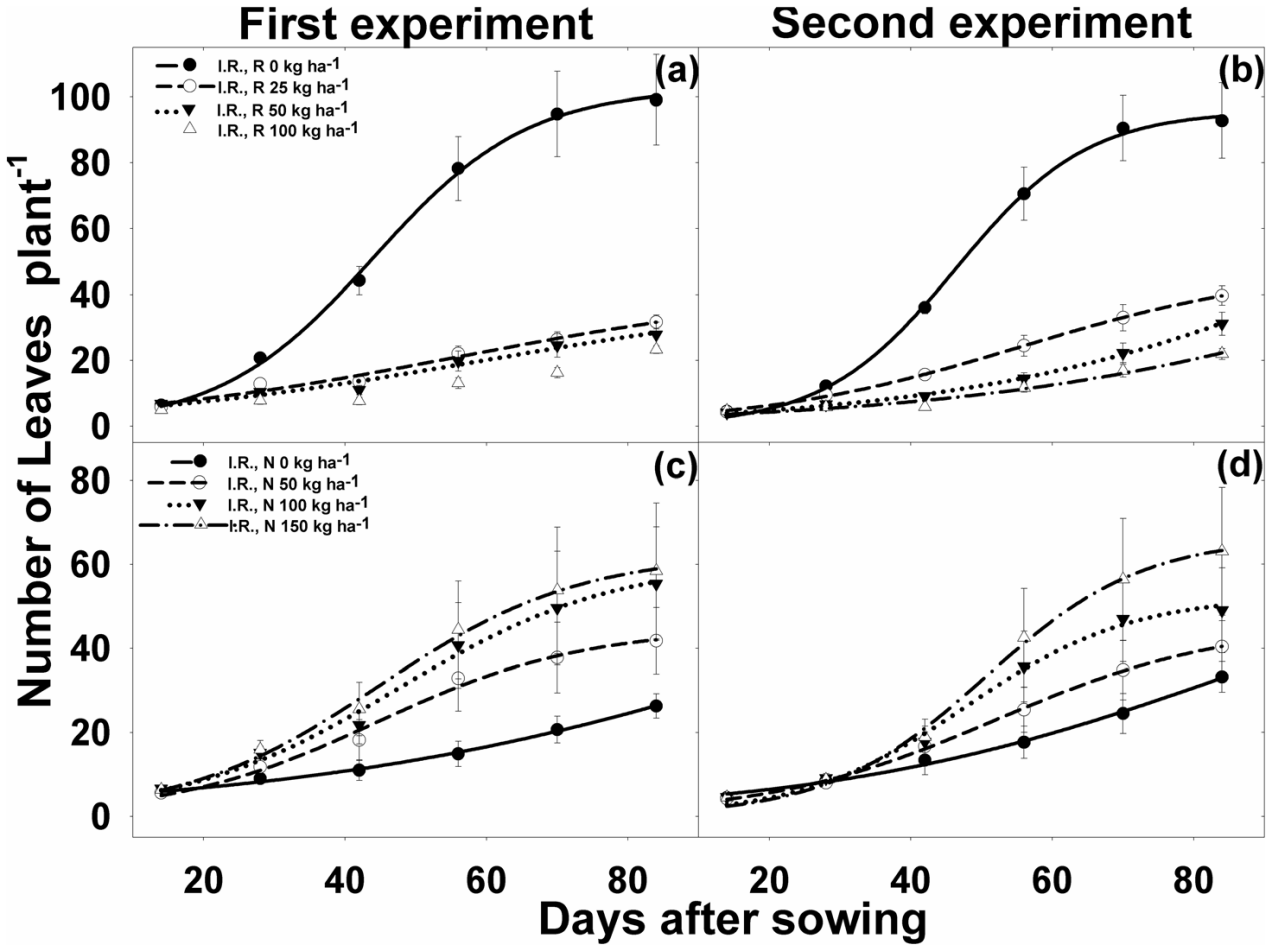
(a, b) Number of leaves per plant of *Ischaemum rugosum* (I.R.), rice seeding rates (R), when grown alone without rice (I.R., R0) or in competition with rice at different seeding rates (R), i.e., 25 (I.R., R25), 50 (I.R., R50), and 100 kg ha^−1^ (I.R., R100); and (c, d) number of leaves per plant when grown under different nitrogen levels, i.e., 0 (I.R., N0), 50 (I.R., N50), 100 (I.R., N100), and 150 kg ha^−1^ (I.R., N150). The vertical bars represent standard error of means. The lines represent a three-parameter sigmoid model (*y* = *a*/{1+e^[−(*x*−*x*50)/*b*]^}) fitted to the data. Parameter estimates are shown in Table 1.

The number of *I. rugosum* leaves per plant declined significantly with the increase in rice seeding rates from 25 to 100 kg ha^−1^ (Figures 4a and b). In the first experiment, rice seeding rates of 25, 50, and 100 kg ha^−1^ reduced the leaf number of *I. rugosum* by 68%, 72%, and 77%, respectively, compared with the leaf number in weeds grown alone (Table 1). A similar reduction (57–76%) was also noted in the second experiment. The number of leaves significantly increased with increasing N levels after 14 DAS (Figures 4c and d Table 1). On average over seeding densities and compared with 0 kg N ha^−1^, the increasing level of N (50, 100, and 150 kg N ha^−1^) increased leaf number by 59%, 111%, and 122%, respectively, in the first experiment; and 22%, 48%, and 90%, respectively, in the second experiment.

### Leaf area

**Figure 5:**
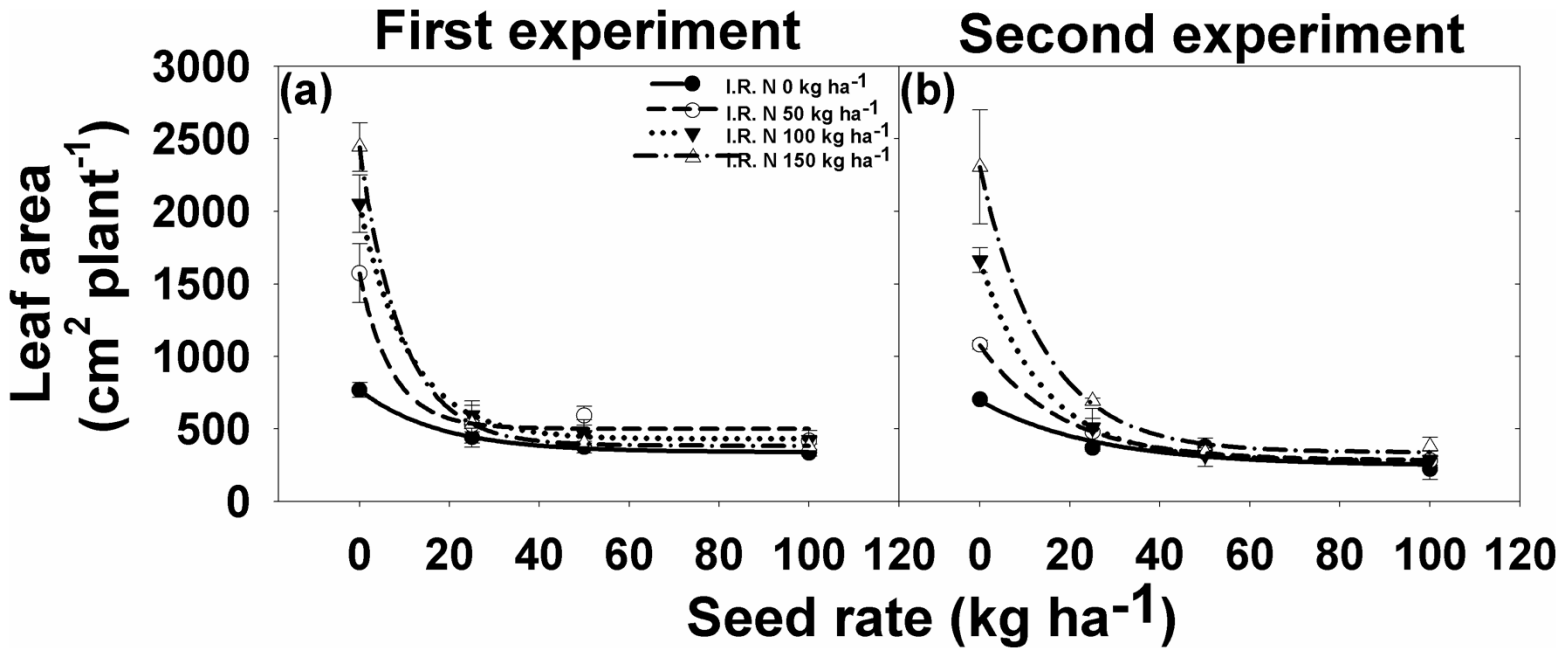
(a, b) Leaf area of *Ischaemum rugosum* under different seeding rates and nitrogen levels. The vertical bars represent standard error of means. The lines represent an exponential model fitted to the leaf area data. Parameter estimates are shown in Table 2.

Interaction between different rice seeding rate and N levels for leaf area plant^−1^ was significant for both the experiments. With increase in rice seeding rate there was a significant reduction in leaf area of *I. rugosum* (Figure 5a Table 2). Compared to the leaf area of weed plants grown without rice competition, rice seeding rates of 25, 50, and 100 kg ha^−1^ reduced leaf area by 69%, 73%, and 77%, respectively (Figure 5a). Leaf area increased with rising levels of N (Figure 5a Table 2). At each rice seeding rate, leaf area (cm^2^ plant^−1^) was always lower in the absence of added N than where N was applied, whereas 150 kg N ha^−1^ produced maximum leaf area (2,442 cm^2^ plant^−1^). In the first experiment, there was an increase of 105% (1,073+501 cm^2^ plant^−1^), 167% (1,622+431 cm^2^ plant^−1^), and 218% (2058+385 cm^2^ plant^−1^) leaf area with added N of 50, 100, and 150 kg ha^−1^, respectively, compared to 0 kg N ha^−1^ (430+339 cm^2^ plant^−1^) (Figure 5a Table 2). A similar trend (55–232% increase) was observed in the second experiment (Figure 5b Table 2).

**Table 2:** The effect of different seeding rates and nitrogen levels on the leaf area of *Ischaemum rugosum*.

N (kg ha^−1^)	First experiment				Second experiment			
	*y0*	*A*	*B*	*R^2^*	*y0*	*a*	*B*	*R^2^*
0	338.7(10.5)	429.8(15.1)	0.06 (0.01)	0.99	244.9 (107.1)	449.9 (134.2)	0.04 (0.03)	0.99
50	501.1(89.0)	1072.8 (150.7)	0.14 (0.18)	0.99	282.4 (4.9)	796.4 (7.0)	0.06 (0.00)	0.99
100	431.0(10.3)	1622.0 (16.6)	0.09 (0.00)	0.99	282.1 (0.3)	1380.9 (0.4)	0.07 (0.01)	0.99
150	384.5 (6.6)	2058.4 (10.8)	0.11 (0.00)	0.99	338.9 (60.4)	1968.6 (92.5)	0.07 (0.01)	0.99

Leaf area data were fitted to an exponential model: *l* = *y0*+*a*×*e^−bx^*, where *l* is the leaf area (cm^2^ plant^−1^) at different seed rates (kg ha^−1^) *x*, *y0* is the minimum leaf area at seed rate 100 kg ha^−1^, *y0*+*a* is the maximum leaf area at seed rate 0 kg ha^−1^, and *b* is the slope. Parameter estimates are followed by standard error in parentheses.

### Leaf biomass

**Figure 6:**
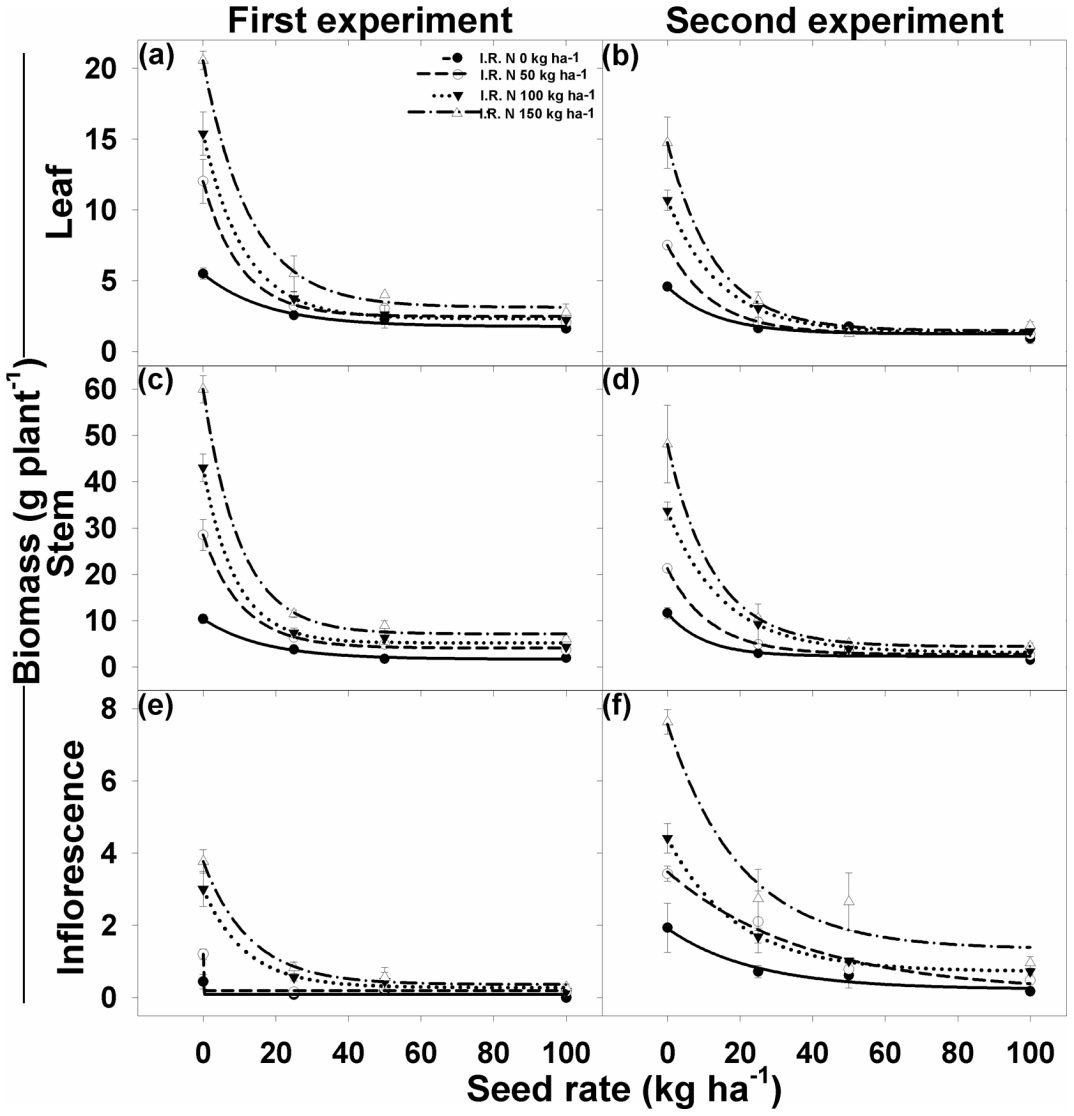
(a, b) Leaf biomass, (c, d) stem biomass, and (e, f) inflorescence biomass of *Ischaemum rugosum* under different seeding rates and nitrogen levels. The vertical bars represent standard error of means. (g) Stem biomass. The lines represent an exponential model fitted to the seedling biomass data.

At each rice seeding rate, the leaf biomass of *I. rugosum* was always lower in the absence of added N in comparison to the treatments where N was applied (Figure 6 Table 3). Interaction between seeding rate and N levels for leaf biomass area plant^−1^ was significant for both the experiments The application of 150 kg N ha^−1^ produced maximum leaf biomass (20.6 g plant^−1^). In the first experiment, there was an increase of 119%, 180%, and 284% leaf biomass with N rates of 50, 100, and 150 kg ha^−1^, respectively, compared to 0 kg N ha^−1^(Figures 6a and b Table 3). A similar response was observed for the second experiment, in which leaf biomass decreased with the increase in rice seeding rate. Compared with the weed plants grown without rice competition, leaf biomass was reduced by 72%, 77%, and 84% with rice seeding densities of 25, 50, and 100 kg ha^−1^, respectively.

**Table 3:** The effect of different seeding rates and nitrogen levels on leaf, stem, inflorescence, and root biomass (g plant^−1^) of *Ischaemum rugosum*.

	N (kg ha^−1^)	*Ischaemum rugosum* biomass (g plant^−1^)											
		Leaf				Stem				Inflorescence			
		*y0*	*a*	*b*	*R^2^*	*y0*	*a*	*B*	*R^2^*	*y0*	*a*	*B*	*R^2^*
First experiment	0	1.77 (0.32)	3.72 (0.47)	0.06 (0.02)	0.99	1.67 (0.40)	8.70 (0.59)	0.06 (0.01)	0.99	0.09 (0.00)	0.35 (0.00)	9964.89 (+inf)	0.84
	50	2.48 (0.51)	9.55 (0.83)	0.10 (0.04)	0.99	4.07 (0.55)	24.44 (0.88)	0.09 (0.02)	0.99	0.19 (0.00)	1.01 (0.00)	808.97 (+inf)	0.99
	100	2.32 (0.13)	13.07 (0.20)	0.09 (0.01)	0.99	5.18 (0.97)	37.84 (1.61)	0.11 (0.03)	0.99	0.28 (0.14)	2.73 (0.23)	0.09 (0.03)	0.99
	150	3.12 (0.53)	17.44 (0.83	0.08 (0.01)	0.99	7.14 (1.39)	52.85 (2.26)	0.11 (0.02)	0.99	0.37 (0.12)	3.41 (0.19)	0.08 (0.02)	0.99
Second experiment	0	1.24 (0.48)	3.33 (0.74)	0.07 (0.06)	0.96	2.30 (0.99)	9.32 (1.61)	0.10 (0.08)	0.96	0.23 (0.25)	1.69 (0.32)	0.04 (0.02)	0.97
	50	1.26 (0.07)	6.25 (0.11)	0.08 (0.01)	0.99	2.69 (0.26)	18.55 (0.42)	0.08 (0.01)	0.99	0.14 (0.64)	3.35 (0.67)	0.03 (0.01)	0.98
	100	1.32 (0.16)	9.38 (0.24)	0.07 (0.01)	0.99	3.11 (0.53)	30.62 (0.79)	0.07 (0.01)	0.99	0.72 (0.04)	3.69 (0.06)	0.05 (0.00)	0.99
	150	1.47 (0.52)	13.30 (0.81)	0.08 (0.02)	0.99	4.40 (0.10)	43.75 (0.16)	0.08 (0.00)	0.99	1.34 (0.99)	6.23 (1.36)	0.05 (0.03)	0.96

Data were fitted to an exponential decay model: *Y* = *y0*+*a*×*e^−bx^* where *Y* is the leaf, stem, and inflorescence biomass (g plant^−1^) at different seed rates (kg ha^−1^) *x*; *y0* is the minimum leaf, stem, and inflorescence biomass at seed rate 100 kg ha^−1^; *y0*+*a* is the maximum leaf, stem, and inflorescence biomass at seed rate 0 kg ha^−1^; and *b* is the slope. Parameter estimates are followed by standard error in parentheses.

### Stem biomass

For stem biomass different rice seeding rate and N levels have significant interaction for both experiments. Regardless of N rate, increasing the seeding rate decreased the stem biomass of *I. rugosum* (Figure 6c). Compared with the plants grown alone, stem biomass decreased by 80%, 85%, and 89% when grown in competition, with rice seeding densities of 25, 50, and 100 kg ha^−1^, respectively. Increasing the level of N increased stem biomass significantly at all seeding rates. In the first experiment, stem biomass of *I. rugosum* increased 2.8, 4.2, and 5.8 times with the application of 50, 100, and 150 kg N ha^−1^, respectively, compared with no added N (Figure 6c Table 3). In the second experiment, the corresponding values were 1.8, 2.9, and 4.1, respectively (Figure 6d Table 3).

### Inflorescence biomass

Different rice seeding rate and N levels have significant interaction for Inflorescence biomass plant^−1^ for both experiments. With the increase in seeding rate, there was a significant decline in the inflorescence biomass of *I. rugosum*, whereas, with the increase in N rate, inflorescence biomass increased significantly (Figures 6e and f Table 3). Compared with the rice seeding rate of 0 kg ha^−1^, the average reduction in inflorescence biomass was 81%, 83%, and 93% for the first experiment (Figure 6e) and 59%, 71%, and 87% for the second experiment (Figure 6f), with rice densities of 25%, 50%, and 100 kg ha^−1^, respectively. Compared to 0 kg N ha^−1^, there was an increase of 173%, 584%, and 759% in the inflorescence biomass with N levels of 50, 100, and 150 kg ha^−1^, respectively, in the first experiment (Figure 6e). In the second experiment, the corresponding values were 82%, 130%, and 294%, respectively (Figure 6f Table 3).

### Total shoot and root biomass

**Figure 7:**
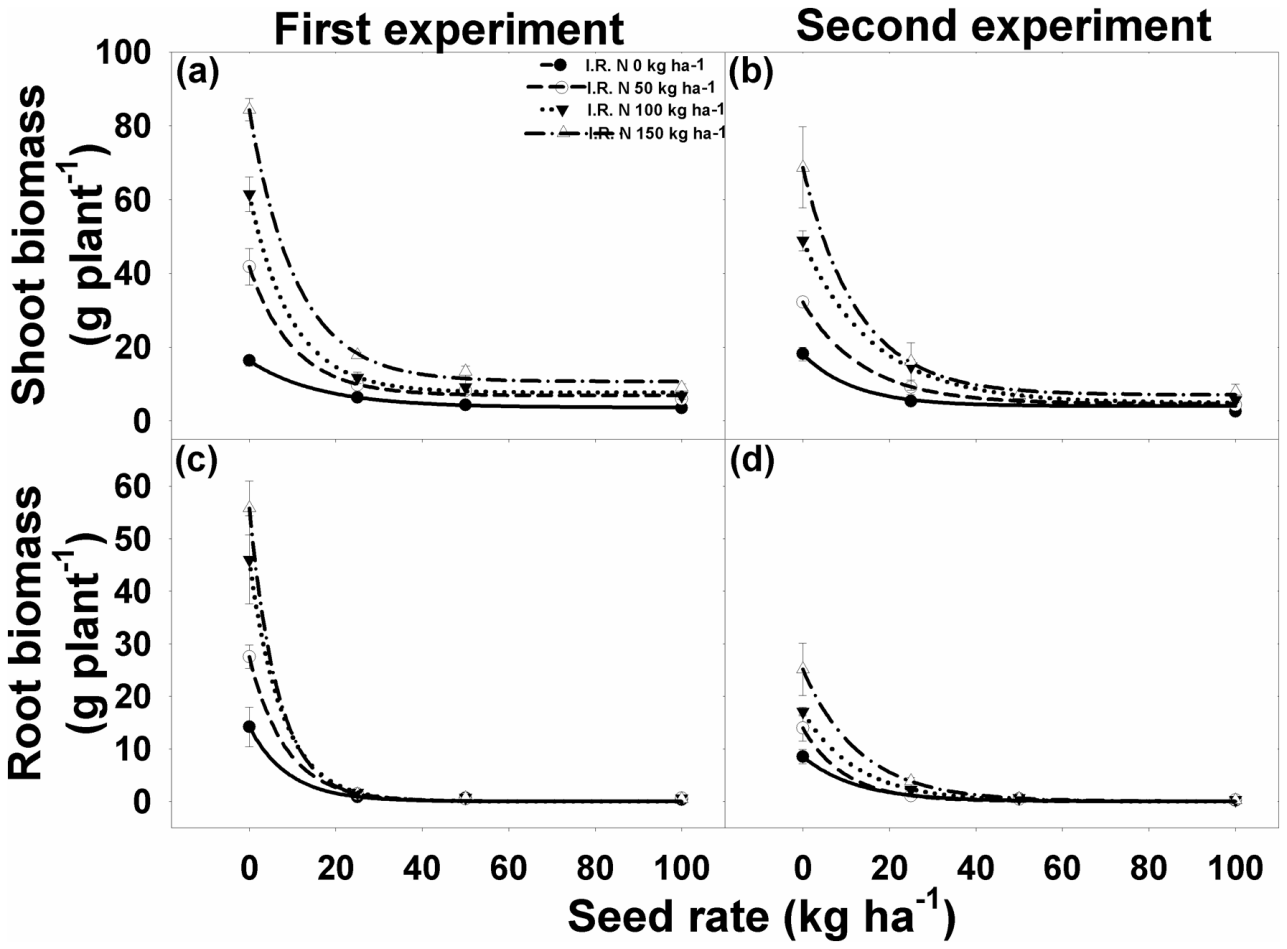
(a, b) Shoot biomass, and (c, d) root biomass of *Ischaemum rugosum* under different seeding rates and nitrogen levels. The vertical bars represent standard error of means. The lines represent an exponential model fitted to the seedling biomass data.

Interaction between different rice seeding rate and N levels for Total shoot and root biomass plant^−1^ was significant for both the experiments. Total shoot and root biomass of *I. rugosum* was affected by rice seeding rates and N levels (Figure 7). With increased seeding rate, there was a decline in shoot and root biomass of *I. rugosum*. Shoot and root biomass decreased by 78% to 99% with rice seeding rates of 25 to 100 kg ha^−1^ compared with the biomass of plants grown alone. The rising level of N increased the shoot and root biomass of both rice and weeds significantly (Figures 7a–d Tables 4 and 5). Results revealed that the effect of N levels on shoot and root biomass of *I. rugosum* was more prominent when weed plants were grown alone than they were grown with rice interference. Increasing levels of N increased the biomass of rice more than it did the weeds (Table 4).

**Table 4:** The effect of different seeding rates and nitrogen levels on aboveground shoot and root biomass (g plant^−1^) of *Ischaemum rugosum*.

Experiment	N (kg ha^−1^)	*Ischaemum rugosum* biomass (g plant^−1^)						
		Shoot				Root		
		*y0*	*a*	*b*	*R^2^*	*A*	*b*	*R^2^*
First	0	3.59 (0.17)	12.70 (0.25)	0.06 (0.00)	0.99	14.20 (0.51)	0.11 (0.02)	0.99
	50	6.82 (1.16)	34.92 (1.89)	0.10 (0.03)	0.99	27.55 (0.56)	0.12 (0.02)	0.99
	100	7.66 (1.11)	53.74 (1.82)	0.10 (0.02)	0.99	45.97 (0.60)	0.13 (0.01)	0.99
	150	10.64 (2.02)	73.68 (3.26)	0.09 (0.02)	0.99	55.85 (0.56)	0.15 (0.02)	0.99
Second	0	3.95 (1.85)	14.16 (2.93)	0.08 (0.07)	0.96	8.55 (0.29)	0.08 (0.01)	0.99
	50	4.59 (0.49)	27.59 (0.75)	0.07 (0.01)	0.99	13.99 (0.31)	0.10 (0.01)	0.99
	100	4.86 (1.24)	44.02 (1.83)	0.06 (0.01)	0.99	17.09 (0.34)	0.08 (0.01)	0.99
	150	7.03 (1.27)	61.69 (1.98)	0.08 (0.01)	0.99	25.17 (0.18)	0.08 (0.00)	0.99

Shoot data were fitted to an exponential decay model: *Y* = *y0*+*a*×*e^−bx^*, where *Y* is the shoot biomass (g plant^−1^) at different seed rates (kg ha^−1^) *x*, *y0* is the minimum shoot biomass at seed rate 100 kg ha^−1^, *y0*+*a* is the maximum shoot biomass at seed rate 0 kg ha^−1^, and *b* is the slope. Root biomass data were fitted to an exponential decay model: *Y* = *ae^−bx^*, where *Y* is the root biomass (g plant^−1^) at seed rate *x* kg ha^−1^, *a* is the maximum root biomass (g plant^−1^) at seed rate 0 kg ha^−1^, and *b* is the slope. Parameter estimates are followed by standard error in parentheses.

**Table 5:** Data on rice tillers per unit area (25 cm^2^); biomass of rice shoot, root, and total plant and root weight ratio (RWR) of the rice plant under different seeding rates, i.e., 0, 25, 50, or 100 kg ha^−1^; and under different nitrogen levels, i.e., 0, 50, 100, and 150 kg ha^−1^ for both experiments.

Seed rate (kg ha^−1^)	First experiment					Second experiment				
	Biomass (g pot^−1^)			Tillers plant^−1^	RWR (g g^−1^)	Biomass (g pot^−1^)			Tillers plant^−1^	RWR (g g^−1^)
	Shoot	Root	Total plant			Shoot	Root	Total plant		
25	39.9	13.8	53.7	19.3	0.381	34.16	11.78	45.94	16.42	0.3529
50	49.5	15.79	65.3	28.6	0.371	41.24	12.28	53.52	23.58	0.3088
100	46.7	15.54	62.3	30.9	0.362	46.56	13.06	59.12	29	0.2775
*S.E.*	4.47	1.20	5.18	2.92	0.032	1.24	0.96	1.98	1.034	0.03
*P*	0.111	0.216	0.09	0.002	0.843	<0.001	0.716	<0.001	<0.001	0.011
N rate (kg ha^−1^)										
0	15.3	7.67	23.0	15.8	0.512	23.45	7.36	30.14	18.11	0.3201
50	37.2	12.46	49.6	22.1	0.340	38.06	12.73	50.79	22	0.3486
100	56.2	17.03	73.2	31.3	0.313	45.74	12.28	58.01	24.89	0.2707
150	72.8	23.02	95.8	35.9	0.320	55.37	17.13	72.50	27.00	0.3130
*S.E.*	5.16	1.38	5.98	3.37	0.037	1.65	1.11	2.29	1.20	0.03
*P*	<0.001	<0.001	<0.001	<0.001	<0.001	<0.001	<0.001	<0.001	<0.001	0.052

### Relationship between rice and weed biomass

**Figure 8:**
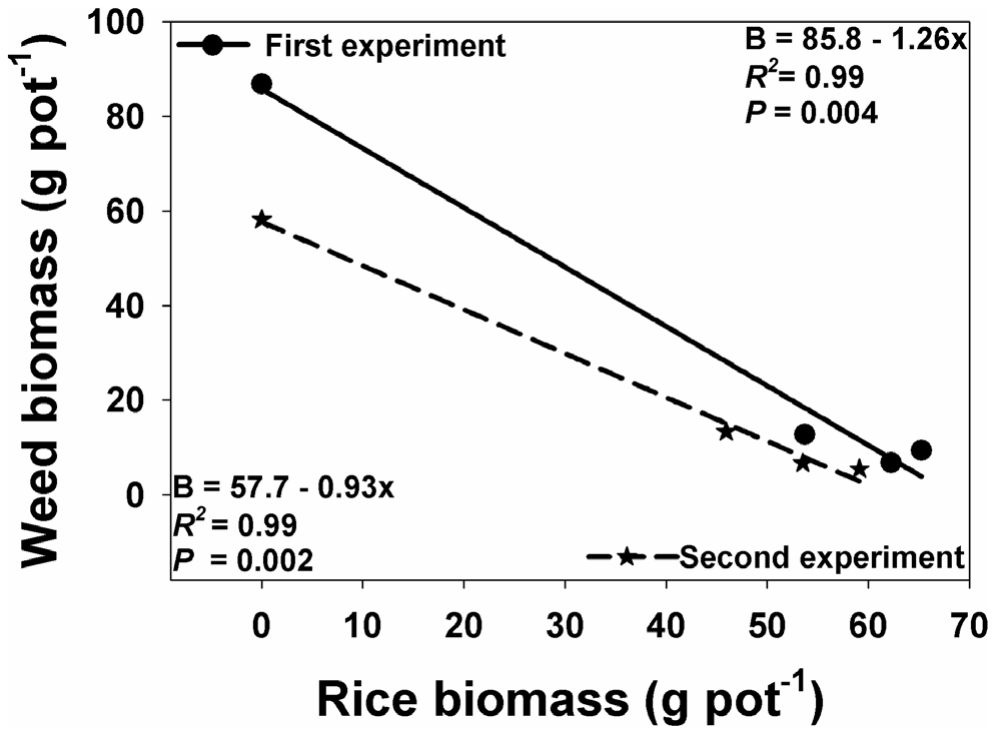
Relationship between rice and *Ischaemum rugosum* biomass (g pot^−1^).

Total plant biomass of rice increased with the increase in rice densities and N levels (Table 5), which suppressed the biomass of *I. rugosum* (Figure 8). The increased seeding rate of rice from 0 to 100 kg ha^−1^ reduced (p<0.001) the total weed biomass, whereas the increasing rate of N increased (p<0.001) the biomass of *I. rugosum*. Compared with the plants grown alone, the biomass of *I. rugosum* was reduced by 85%, 89%, and 92% for the first experiment, and 77%, 89%, and 91% for the second experiment with seeding rates of 25, 50, and 100 kg ha^−1^, respectively. There was an increase in *I. rugosum* biomass by 106%, 196%, and 293% for the first experiment and 59%, 125%, and 206% for the second experiment at N rates of 50, 100, and 150 kg ha^−1^, respectively, compared with the no N-added treatment.

Over the average of two experiments, compared with no added N, increase in weed biomass was 82%, 160%, and 150% and increase in rice biomass was 92%, 155%, and 229% with the addition of 50, 100, and 150 kg N ha^−1^.

Correlation and regression analysis between the biomass of rice and weeds showed that every unit increase in rice biomass decreased weed biomass by 1.25 times in the first experiment and 0.93 times in the second experiment (Figure 8).

### Leaf area ratio

**Figure 9:**
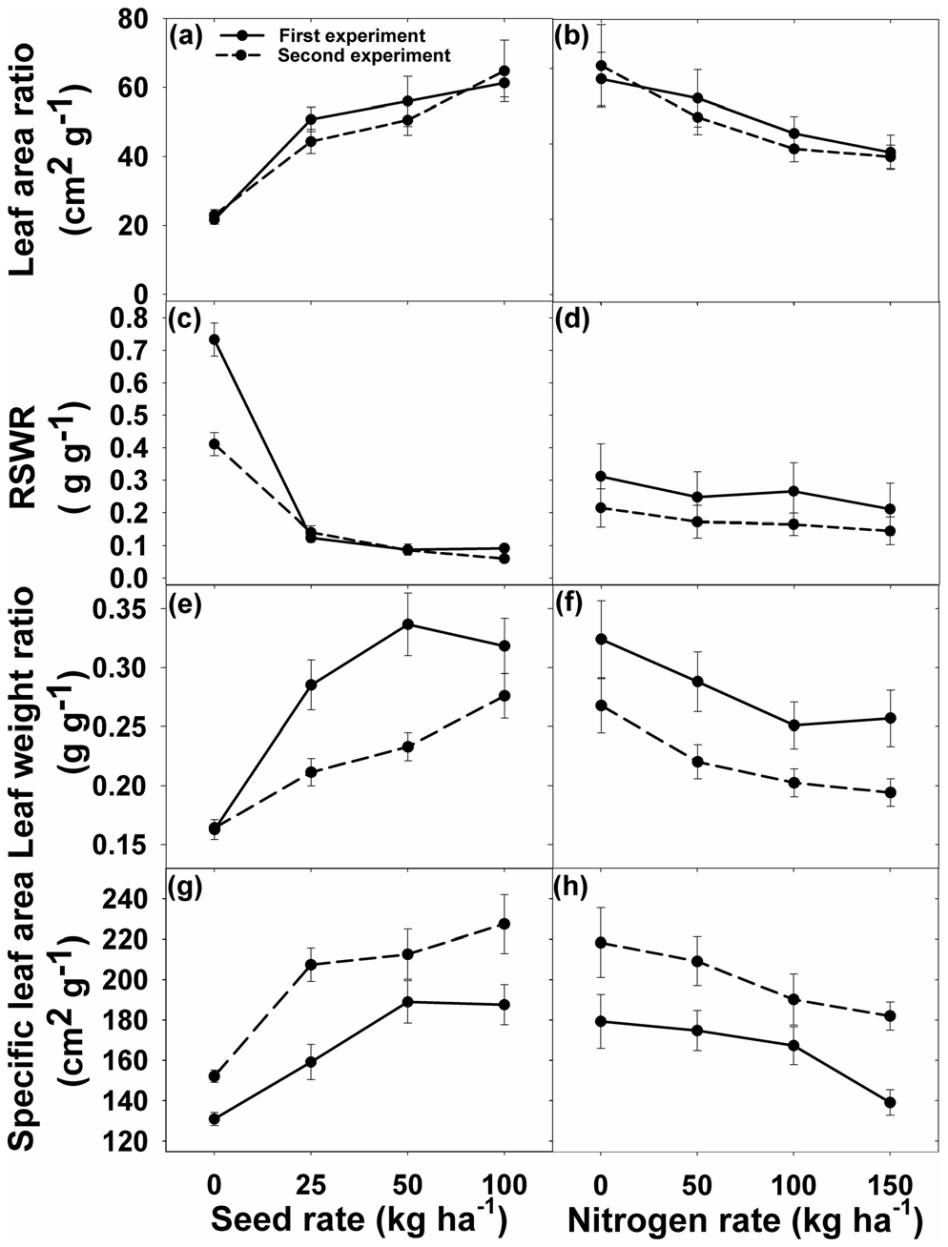
(a, b) Leaf area ratio (LAR); (c, d) root-to-shoot weight ratio (RSWR); (e, f) leaf-weight ratio (LWR); and (g, h) specific leaf area (SLA) of *Ischaemum rugosum* under different seeding rates and nitrogen levels. The vertical bars represent standard error of means.

With the increase in seeding rate, there was an increasing (p<0.001) trend in leaf area ratio (LAR) irrespective of N rates (Figure 9a). At each rice seeding density, LAR decreased (p = 0.006 and 0.003 for the first and second experiments, respectively) with the increase in N for both experiments. For the first experiment, the increase in LAR was 234%, 258%, and 283% at rice densities of 25, 50, and 100 kg ha^−1^, respectively, compared to 0 kg ha^−1^. In contrast, compared with 0 kg N ha^−1^, there was a decrease in LAR by 9%, 25%, and 34% at N rates of 50, 100, and 150 kg ha^−1^, respectively. Similarly, LAR increased with increasing seeding rates and decreased with increasing N rates in the second experiment (Figure 9b).

### Root to shoot weight ratio

The interaction between seeding and N rates was not significant for root to shoot weight ratio (RSWR). Seeding rate had a significant effect (p<0.001), whereas N rate had a non-significant effect on RSWR in both experiments (Figure 9c). RSWR was highest (0.733 and 0.312 in the first and second experiments, respectively) when weed plants were grown alone and the ratio reduced to 0.09 and 0.21 for the first and second experiments, respectively, when weed plants were grown with rice plants at 100 kg seed ha^−1^ (Figure 9d).

### Leaf weight ratio

Increased seeding rate from 0 to 100 kg ha^−1^ increased (p<0.001) leaf weight ratio (LWR) of *I. rugosum* (Figure 9e). Compared with the plants grown alone, LWR increased by 195%, 181%, and 199% for the first experiment and 129%, 135%, and 169% for the second experiment at rice seeding rates of 25, 50, and 100 kg ha^−1^, respectively (Figure 9e). Compared to 0 kg N ha^−1^, the increased levels of N (50–150 kg ha^−1^) reduced LWR by 11–21% for the first experiment and 18–28% for the second experiment (Figure 9f).

### Specific leaf area

In both experiments, increase in the rice seeding rate increased (p<0.001) the specific leaf area (SLA) of *I. rugosum*, whereas N rates did not affect the SLA of the weeds. Compared with the plants grown alone, increase in SLA was 122%, 144%, and 143% for the first experiment, and 147%, 146%, and 161% for the second experiment at rice seeding rates of 25, 50 and 100 kg ha^−1^, respectively (Figure 9g). Compared with the treatment without added N, the decrease in SLA was 3%, 7%, and 23% for the first experiment and 4%, 13%, and 17% for the second experiment at N rates of 50,100, and 150 kg ha^−1^, respectively (Figure 9h).

## Discussion

The height of *I. rugosum* was affected by high rice seeding rates and N rates. N application had a more positive effect on the rice plant height than it had on the weed. N effect was more pronounced on the height of *I. rugosum* plants grown alone compared to the plants grown in competition with rice. Similar findings were reported in an early study, in which the growth of *Equistem arvense* L. increased at high rates of N supplied [22]. With the application of N, *I. rugosum* growth can be suppressed by rice interference. *Ischaemum rugosum* was always taller than rice owing to the reason that the weed has a pronounced shade-avoiding syndrome. With increasing seeding rates (0–100 kg ha^−1^), weed height increased, irrespective of N levels, to overcome the effects of shade for capturing sunlight for photosynthesis [23]. Similar results were reported for *Echinochloa phyllopogon* (Stapf) Koss., whose height increased with the increase in shade [21].

With increasing rice seeding rate, there was a decline in the biomass of aboveground (stem, leaves, and inflorescence) and belowground (root) plant parts of *I. rugosum*. Results are in line with an earlier study on *E. crus-galli*, in which aboveground biomass decreased (38–48%) significantly with increasing rice density and increased with the increase in N rates [24]. Earlier, researchers reported that *E. phyllopogon* biomass decreased under shaded conditions and increased with the increase in N rates from 0 to 224 kg ha^−1^ [21]. In our study, as the shading by high rice density increased, root biomass decreased. This proved that under the sunniest environments, the roots of plants preferred sinks for photosynthates, whereas under the shadiest circumstances, the shoots were the most important sinks during almost the entire life of the plant [25].

Increasing N rates did not increase *I. rugosum* biomass at the same rate as of rice biomass. In earlier studies in corn [16] and soybean [26] the adverse effects of weed competition was severe on crops at low N rates than high N rates. Soybean was more responsive to N than weeds, which indicates that high N rate is more beneficial to crops than weeds. At initial crop growth stages, low N rate make crops vulnerable to weed competition. Higher N rates can enhance rapid crop growth and canopy closure, required to suppress the growth of weeds [26]. Thus, rice-weed interactions can be strongly affected by N availability [14], [21].

With the increase in seeding rate, there was a reduction in leaf area and leaf biomass of *I. rugosum* irrespective of N rates; whereas, with the increase in N rate, there was an increase in leaf area and leaf biomass. This increase can be attributed to changes in total biomass. Total plant or shoot biomass increased with the increase in N and this is highly correlated with leaf area. Similar results were also reported for *E. phyllopogon*, in which leaf area increased as N increased from 0 to 224 kg ha^−1^ because more biomass was available for allocation to leaves [21]. In *C. selloana*, leaf area per plant was five times higher where N was applied as compared to the control [27].

LAR and RSWR indicate how photosynthates were being apportioned among the plant parts. LAR and SLA also demonstrate the utilization of photosynthates available for the growth of leaves (leaf expansion vs. leaf thickness). Results revealed that higher seeding rates increased LAR, LWR, and SLA and reduced RSWR, whereas higher N rates reduced LAR, LWR, SLA, and RSWR. *Ischaemum rugosum* plants grown with rice at 100 kg seed ha^−1^ (shade) had a lower RSWR and higher LAR, LWR, and SLA than plants grown without rice interference (no shade), indicating that the weed has a phenotypic plasticity to allocate more photosynthates to aboveground plant parts than those belowground when grown in competition. It seems that partitioning of biomass in *I. rugosum* was affected more by limitations in light than by N availability. Weed RSWR decreased more than rice RSWR with increase in rice seeding rates (shade) and N rates. Increased partitioning to roots is a common compensatory response of plants to low N availability [21]. Reynolds and Antonio made 77 studies representing 129 species. They found that RSWR decreased with increase in N availability [28]. Bonifas *et al.* reported that RSWR decreased for *Abutilon theophrasti* Medik. and corn with increase in N application [29].

The production of higher SLA by the weed under high rice seeding rates demonstrates that *I. rugosum* has shade-avoiding syndrome characteristics and produced thinner leaves to avoid the shading effects of rice leaves [30]. This weed produced thinner leaves to increase leaf area per allocated photosynthates to intercept light and maintain photosynthesis, which are crucial for the life of plants. Thinner leaves enhanced the amount of radiation absorbed per chloroplast in the leaf to increase the rate of photosynthesis [31]. *Ischaemum rugosum* was able to change leaf thickness to adapt to reduction in sunlight by producing thin leaves under shady conditions and thicker leaves under full light conditions. This phenotypic plasticity made the weed extremely competitive with rice.

## Conclusions and Implication of the study

*Ischaemum rugosum* showed plasticity in SLA, LAR, LWR, and RSWR in response to competition with rice plants. Increase in rice seeding rates increased SLA, LAR, and LWR and reduced RSWR, whereas all these ratios declined with increase in N rate. *Ischaemum rugosum* plants are phenotypically plastic and can alter their morphology and physiology to improve the acquisition of a resource, when lack of that resource limits the growth of the plant [21]. Increased partitioning of biomass to shoot vs. roots (low RSWR) is a common response of shade-avoiding plants to shade and, in addition to physiological and morphological changes in leaf (increased LAR, LWR, and SLA), these plants can reduce the effects of shade. The weed had a higher capacity for N uptake compared to rice plants. But when the weed was grown with rice interference, rice plants caused reduced N uptake of the weed (data not shown).

Our study suggests that management strategies that depend on shade due to crop interference may not provide complete suppression of *I. rugosum*. This was highlighted by observations on its plant height, LAR, LWR, SLA, RSWR, and inflorescence biomass when grown alone or in competition with a varying density of rice plants. Although *I. rugosum* cannot be completely suppressed by crop interference, high seeding rates can reduce the biomass of *I. rugosum* appreciably and make it weak and vulnerable and thus can be easily controlled. This study was conducted in controlled conditions and the results can suggest possile approaches for weed management that are worthy of corroboration by further field study. However, the results of this study can be used to aid the conceptualization of weed management strategies for *Ischaemum rugosum*
